# The relationship between social support and posttraumatic stress symptoms among youth exposed to a natural disaster

**DOI:** 10.1080/20008198.2018.1450042

**Published:** 2018-03-22

**Authors:** Betty S. Lai, Melissa C. Osborne, Jennifer Piscitello, Shannon Self-Brown, Mary Lou Kelley

**Affiliations:** a School of Public Health, Georgia State University, Atlanta, GA, USA; b Department of Psychology, Louisiana State University, Baton Rouge, LA, USA

**Keywords:** Disaster, posttraumatic stress, children, social support, hurricane, desastre, estrés postraumático, niños, apoyo social, huracán, 灾难, 创伤后应激, 儿童, 社会支持, 飓风, • The purpose of this study was to examine bidirectional relationships between social support (from parents, teachers, and peers) and PTSS among a sample of children exposed to Hurricane Katrina. • Models simultaneously tested social causation and social selection models. • To our knowledge, this is the first study to examine these issues among children longitudinally. • Overall, there was support for social selection mechanisms, and limited support for social causation mechanisms.

## Abstract

**Background**: Children are a vulnerable population following a natural disaster, due to their age and dependence on adults. The primary presenting problem children report after disasters is posttraumatic stress symptoms (PTSS). Prior research suggests that PTSS is inversely related to social support, which is often disrupted after a disaster.

**Objective**: This study examined the relationship between social support (from parents, teachers, and peers) and PTSS in children affected by Hurricane Katrina. The research contributes to the literature by examining the mechanisms that drive this relationship over time.

**Methods**: In this study, 426 children were followed over four timepoints, beginning 3–7 months after Hurricane Katrina and concluding 25–27 months post-hurricane. Three path models analysed the relationship between social support (from parents, teachers, and peers, measured by the Social Support Scale for Children) and PTSS (measured by the UCLA PTSD Reaction Index). Covariates included child age, minority status, gender, perceived life threat, and actual life threat. Nonsignificant paths were trimmed from the final models. Global fit indices were examined to determine model fit.

**Results**: In the parent and peer social support models, PTSS exhibited statistically significant effects on social support from one wave to the next. In the teacher model, this was only true between Waves 2 and 3. Social support showed a statistically significant effect on PTSS between Wave 2 and Wave 3 in the peer model (standardized estimate = −0.26, *p* < .0001). No paths from social support to PTSS were significant in the parent and teacher models.

**Conclusion**: Findings support a social selection model in which PTSS undermine social support, particularly in the first two years post-disaster. If these findings are replicated, this suggests that, in cases of limited funding, PTSS should be prioritized, given their cascading effects on social support.

## Abstract (es)

**Planteamiento**: Los niños son una población vulnerable después de un desastre natural, debido a su edad y a su dependencia de los adultos. El principal problema que presentan los niños después de los desastres son síntomas de estrés postraumático (SEPT). La investigación previa sugiere que los SEPT están inversamente relacionados con el apoyo social, que a menudo se ve afectado después de un desastre.

**Objetivo**: Este estudio examinó la relación entre el apoyo social (de padres, maestros y compañeros) y los SEPT en niños afectados por el huracán Katrina. La investigación contribuye a la literatura mediante el examen de los mecanismos en los que se basa esta relación a lo largo del tiempo.

**Métodos**: En este estudio, se siguió a 426 niños a lo largo de cuatro puntos en el tiempo, comenzando 3-7 meses después del huracán Katrina y concluyendo 25-27 meses después del huracán. Tres modelos de pautas analizaron la relación entre el apoyo social (de padres, maestros y compañeros, medido por la Escala de apoyo social para niños) y los SEPT (medido por el índice de reacción de TEPT de UCLA). Las covariables incluyeron la edad del niño, el estatus de minoría, el género, la amenaza de muerte percibida y la amenaza de muerte real. Las pautas no significativas fueron excluidas de los modelos finales. Se examinaron los índices de ajuste global para determinar el ajuste del modelo.

**Resultados**: En los modelos de apoyo social para padres e iguales, los SEPT mostraron efectos estadísticamente significativos en el apoyo social de una onda a la siguiente. En el modelo de maestros, esto solo fue cierto entre las ondas 2 y 3. El apoyo social mostró un efecto estadísticamente significativo en los SEPT entre la onda 2 y la onda 3 en el modelo de iguales (estimación estandarizada = −0.26, *p* < .0001). Ninguna pauta desde el apoyo social a los SEPT fue significativa en los modelos de padres y maestros.

**Conclusión**: Los hallazgos apoyan un modelo de selección social en el que los SEPT socavan el apoyo social, especialmente en los dos primeros años después del desastre. Si se replican estos hallazgos, esto sugiere que, en casos de financiación limitada, se debe priorizar los SEPT, dados sus efectos en cascada sobre el apoyo social.

## Abstract (zh)

**背景：**自然灾害后儿童属于脆弱人群，这是因为儿童的年龄小，并且对成人有依赖性。在灾害后，儿童出现的主要问题是创伤后应激症状（PTSS）。前人研究指出，PTSS 和社会支持负相关，但社会支持经常在灾害后被中断。

**目标：**本研究考察了（来自父母、教师和同伴的）社会支持和飓风Katrina后儿童的PTSS之间的关系。本研究结果有助于了解该关系随时间的变化。

**方法：**426名儿童在四个时间点参加研究，从飓风Katrina的3-7月前到飓风发生的25-27月后。三个路径模型分析了（来自父母、教师和同伴的）社会支持（《儿童社会支持量表》）和 PTSS(《UCLA PTSD 反应指数》)之间的关系。协变量包括儿童年龄，少数群体状态，性别，感知到的生活威胁和实际生活威胁。不显著的路径从最终模型中排除。使用全局拟合指数（Global fit indices ）决定模型拟合性。

**结果：**在父母和同伴社会支持模型中，PTSS 对下一轮社会支持的效应统计显著。在教师模型中，这种效应只出现在第二和第三轮。在同伴模型中，社会支持在第二和第三轮之间显示了对 PTSS 的显著效应（标准化估计值=-0.26, *p*<.0001）。在家长和教师中，社会支持对 PTSS 的路径都不显著。

**结论：**结果支持了社会选择模型：PTSS 损害了社会支持，尤其是在灾后最初两年。如果这些研究发现得到重复验证，这说明在研究资金有限的前提下， PTSS 因其对接下来社会支持的影响，需要被优先考虑。

Climate change is increasing the frequency and intensity of weather-related events (‘After Louisiana’, 2016). Currently, over 100 million children worldwide are exposed to disasters every year (Save the Children, 2007; United Nations International Strategy for Disaster Reduction [UNISDR], 2013). The USA Global Change Research Program (2016) released an assessment of the impact of climate change on human health in the USA. The report highlighted mental health as a primary area impacted by climate change. Children are a vulnerable population post-disaster (Lai, Auslander, Fitzpatrick, & Podkowirow, 2014; Lai, Esnard, Lowe, & Peek, 2016), due to their age and dependence on adults. A large body of literature documents that children exposed to disasters are at risk for developing mental health symptoms (Dogan, 2011; Lai, Alisic, Lewis, & Ronan, 2016; Lai, La Greca, & Llabre, 2014). Posttraumatic stress symptoms (PTSS) are the primary presenting mental health problem among children after disasters (Dyregrov & Yule, 2006; Furr, Comer, Edmunds, & Kendall, 2010; Lai, Kelley, Harrison, Thompson, & Self-Brown, 2015b; Ponnamperuma & Nicolson, 2016). More research is needed that addresses how to mitigate the mental health consequences of disasters for children.

A prime target for child post-disaster interventions is social support (Kassam-Adams, 2014; Reifels et al., 2013). Social support is frequently disrupted for children after disasters (Derivois, Merisier, Cenat, & Castelot, 2014; Gordon-Hollingsworth et al., 2015; Rubens, Vernberg, Felix, & Canino, 2013). At the same time, social support may be an important protective factor that buffers children from experiencing significant symptoms of distress (Jieling & Xinchun, 2017; Ma et al., 2011). Inadequate social support is a significant risk factor for PTSS in response to multiple types of disasters, including floods, tornados, hurricanes, earthquakes, fires, and man-made disasters (Bokszczanin, 2008, 2012; Danielson et al., 2017; La Greca et al., 2013). This is an important association among adults as well, as demonstrated by a meta-analysis of risk factors for Posttraumatic Stress Disorder among adults, which found lack of social support to have stronger effects than pre-trauma factors on Posttraumatic Stress Disorder (Brewin, Andrews, & Valentine, 2000).

Studies are needed that use theory-driven models to test the relationship between social support and PTSS. Among adults, initial research supports both social causation and social selection models (Kaniasty & Norris, 2008). Social causation models posit that lower social support leads to increased risk for mental health problems (Johnson, Cohen, Dohrenwend, Link, & Brook, 1999; Platt, Lowe, Galea, Norris, & Koenen, 2016). For children, this might mean that reduced social support impairs their ability to cope with distress. Conversely, children with greater social support may be less vulnerable to developing distress. Studies indicate that social support may protect children from distress symptoms in the aftermath of disasters (Banks & Weems, 2014; La Greca, Silverman, Lai, & Jaccard, 2010). In contrast, social selection models theorize that psychiatric symptoms undermine social support (Dohrenwend, 2000; Lowe & Willis, 2015). This theoretical framework would indicate that PTSS disrupt children’s engagement with others, diminishing children’s social support.

Despite the critical connection between social support and PTSS, to our knowledge no studies exist at this time that have simultaneously tested these bidirectional mechanisms among children post-disaster. This is due in part because few studies have assessed children at multiple timepoints post-disaster, and even fewer studies have followed children beyond the first 12 months (Kessler, Keane, Ursano, Mokdad, & Zaslavsky, 2008; Pfefferbaum et al., 2014). Studies are needed that simultaneously test these mechanisms in order to understand how and when the relationship between social support and PTSS arises and develops.

Further, to better inform intervention efforts, it is also important to consider the source of social support provided to children after a disaster. For example, social support from parents may be more important than that provided by peers. Moore and Varela (2010) examined social support from parents, teachers, and classmates in 156 children exposed to Hurricane Katrina. They found that classmate support was negatively related to PTSS, but parent and teacher support were unrelated to PTSS. By examining the influence and source of social support in children’s post-disaster recovery, specific educational and training efforts can be designed and implemented that provide educators with the tools necessary to foster and provide social support for children in need. Elucidating the relationship between social support and PTSS will allow for the development of preventative public health efforts to prepare adults, such as parents and teachers, to better promote recovery and resilience in children impacted by natural disasters.

To address these gaps in the literature, the aim of this study was to examine bidirectional relationships between social support (from parents, teachers, and peers) and PTSS among a sample of children exposed to Hurricane Katrina. Children were followed over four timepoints, 3–27 months post-disaster. Social causation and social selection models were simultaneously tested. Due to the varied outcomes in the existing literature on this topic, we expected to find support for both models.

## 1. Method

A sample of 426 children residing in New Orleans and the surrounding areas during the time of Hurricane Katrina participated in the study. Hurricane Katrina was a Category 5 hurricane that made landfall in southern Louisiana in 2005, destroying thousands of homes, businesses, and other properties (Knabb, Rhome, & Brown, 2005). Hurricane Katrina caused an estimated 1500 deaths and US$108 billion damage (Knabb et al., 2005). Participants in the present study were part of a larger multi-wave, longitudinal study investigating the psychological impact of Hurricane Katrina on mothers and their children (Kelley et al., 2010; Lai et al., 2015a, 2015b; Lai, Auslander, Fitzpatrick, & Podkowirow, 2014; Self-Brown, Lai, Thompson, McGill, & Kelley, 2013).

Data were collected across four timepoints post-Hurricane Katrina. Wave 1 was collected at 3–7 months. Wave 2 was collected at 13–17 months. Wave 3 was collected at 19–22 months. Wave 4 was collected 25–27 months.

A majority of children in the sample were displaced after the storm (69%). Over half of participants (55%) reported an annual family household income prior to Hurricane Katrina of less than US$25,000, the lowest income bracket. Further, 56% of participants were children of single parents at Wave 1. Child participants (55% female) ranged in age from 8–16 years (M = 11.62, *SD* = 1.56) and were in the 3rd–8th grades during initial data collection. Approximately three-quarters of the children were members of a racial/ethnic minority group.

### 1.1. Procedure

After obtaining IRB approval from Louisiana State University, six schools were selected as recruitment locations for this study. Parents at the schools were provided with information about the study and were invited to participate. Parents who expressed interest in participating were provided consent and assent forms and study questionnaires. Parents returned completed questionnaires in sealed envelopes to their child’s school or mailed them in pre-paid envelopes provided by the study investigators. Children completed their questionnaires in small groups at their school. Trained undergraduate and graduate student research assistants administered questionnaires. At the first timepoint, students were compensated at the discretion of each of the participating schools (e.g. pizza party drawing or US$50 prize drawing). At subsequent timepoints (i.e. Waves 2–4), parents were compensated US$25–50 and children received small items (e.g. stickers or pencils).

Of those who were invited to participate, 35% consented and completed questionnaires. At Wave 1, 388 children participated. An additional 38 children joined the study at Wave 2, and a total of 367 children completed the assessment. At Wave 3, 348 children participated. At Wave 4, 333 children participated. There were 235 children (55%) who participated in all four waves of data collection. An additional 41 participants completed measures at Waves 1 and 2 as well as at either Wave 3 or Wave 4.

### 1.2. Measures

Mothers (*n* = 424) or fathers (*n* = 2) completed the demographic questionnaire. Children completed all other measures used in this study.

#### 1.2.1. Demographic questionnaire

A demographic questionnaire completed by parents was used to obtain descriptive information about children’s gender (male or female), age, race, parents’ marital status, and family annual income. Child age was captured as a continuous variable. Race was a categorical variable with seven categories. However, for the analysis, a binary minority status variable was created whereby children who were not identified as Non-Hispanic white were considered to have minority status. Parents’ marital status and family annual income were included for descriptive purposes in this study but were not included as covariates in the models; they were categorical variables with five categories for marital status and nine categories for income in increments of US$5000.

#### 1.2.2. Hurricane related traumatic experiences

This 15-item child-report questionnaire was adapted from that used in similar studies assessing hurricane loss and exposure in youth samples (La Greca, Silverman, & Wasserstein, 1998; Vernberg, La Greca, Silverman, & Prinstein, 1996). The Hurricane Related Traumatic Experiences questionnaire included assessments of perceived (1 item) and actual life-threatening experiences (6 items). Example actual life threat items include: ‘Did windows or doors break in the place you stayed during the hurricane’ and ‘Were your toys or clothes ruined by the hurricane?’ To calculate an actual life threat score, the six items were summed. Mean actual life threat scores reflect the mean of the sum of the actual life threat summary score. Perceived life threat is measured through the single question, ‘At any time during the hurricane, did you think you might die?’ Response options for both perceived and actual life threat items included ‘no’ (scored as 0) or ‘yes’ (scored as 1) for each question. Scores collected from the children at Wave 1 were used in the analysis. This measure has been used in previous investigations to assess disaster exposure in children (La Greca et al., 2010; Lai et al., 2015a, 2015b; Lai, La Greca, Auslander, & Short, 2013).

#### 1.2.3. Social Support Scale for Children (SSSC; Harter, 1985)

The SSSC is a widely used measure that assesses perceived social support from four sources: parents, teachers, friends, and classmates. For this study, support from friends and classmates were collapsed into one social support category referred to as peer. Thus, three sources of social support were assessed in this study. This 24-item child-report measure includes two choices per item. First, children select a statement that they most identify with. For instance, ‘Some kids have parents who treat their child like a person who really matters’ or ‘Other kids have parents who don’t usually treat their child like a person who really matters’. Each item contains two statements structured in this manner. Once the child selects the statement he or she most identifies with, the child rates the statement as either being ‘really true for me’ or ‘sort of true for me’. Responses are coded from 1 to 4, depending on the statement and response selected, with higher scores indicating greater support. See Table 1 for sample summary statistics for this measure. This scale demonstrates good psychometric properties (Harter, 1985; Lipski, Sifers, & Jackson, 2014). Internal consistency across all timepoints was adequate with Cronbach’s alpha ranging from 0.76 (Wave 1) to 0.83 (Wave 4) for parental support; 0.69 (Wave 1) to 0.82 (Wave 3) for teacher support; and 0.82 (Wave 1) to 0.87 (Wave 4) for peer support.

**Table 1.** Sample descriptive statistics for study variables, means (*SD*).

Child characteristic	Wave 1 (*n* = 388)	Wave 2 (*n* = 367)	Wave 3 (*n* = 348)	Wave 4 (*n* = 333)
Parent Social Support Mean (Range 1–4)	3.34 (0.66)	3.45 (0.63)	3.49 (0.64)	3.52 (0.60)
Teacher Social Support Mean (Range 1–4)	3.18 (0.61)	3.22 (0.65)	3.27 (0.70)	3.34 (0.61)
Peer Social Support Mean (Range 1–4)	3.12 (0.60)	3.25 (0.61)	3.35 (0.57)	3.38 (0.56)
Post-Traumatic Stress Score Mean (Range 0–4)	1.08 (0.87)	0.86 (0.78)	0.75 (0.75)	0.63 (0.66)

#### 1.2.4. Posttraumatic stress symptoms

UCLA-PTSD Reaction Index-Revision 1 (UCLA-PTSD-RI-R1; Pynoos, Rodriguez, Steinberg, Stuber, & Frederick, 1998). The UCLA-PTSD RI-R1 is an 18-item child-report scale assessing 17 symptoms of PTSD as per DSM-IV-TR (American Psychiatric Association, 2000) in children aged 6–17. This measure has been used in samples of children who were exposed to disaster-related events (Weems et al., 2010; Yelland et al., 2010). Children were asked to rate their symptoms based on their experience related to Hurricane Katrina. They rated symptoms on a 5-point scale ranging from ‘none of the time’ to ‘most of the time’. A sample item for this measure includes: ‘Do you ever get scared, afraid, or upset when you think about [event]?’ The higher score on item 10 or item 11 (measuring emotional numbness) was used in computing the scale scores. Mean scores (ranging from 0–4) were used for the purposes of this study rather than summary scores. Internal consistency across all timepoints was excellent with Cronbach’s alpha ranging from 0.91 (Wave 2) to 0.93 (Wave 3).

### 1.3. Statistical analysis

Descriptive statistics were analysed using SPSS version 23 (IBM Corp, 2015). The primary analyses were run in Mplus version 8 (Muthén & Muthén, 1998–2017). Missing data were treated using full information maximum likelihood (FIML) approach, as data met the assumption for missing at random (MAR). Specifically, with regard to missing data, 61 (14%) participants dropped out of the study by Wave 3. Statistical tests (*t*-tests and Chi-square tests, as appropriate) were conducted to assess differences between those who completed the study and those who dropped out. These tests found no statistically significant differences between the two groups. Mean scores for PTSS and social support were used. Regarding missing data, 81 participants were missing PTSS means for Wave 1, 71 for Wave 2, 82 for Wave 3, and 96 for Wave 4. For mean social support, across the three sources examined, 108 participants were missing data at Wave 1, 79 at Wave 2, 89 at Wave 3, and 100 at Wave 4.

Cross-lagged models were based on those developed by Platt et al. (2016), who examined social selection and social causation mechanisms among adults after Hurricane Ike. Path models were developed for the primary analyses using mean scores for the PTSS and social support variables. Model fit was assessed using global fit indices (i.e. chi-square test of model fit, Root Mean Square Error of Approximation, Comparative Fit Index, Standardized Root Mean Square Residual) as well as modification indices and standardized residuals. Each model was run separately for parent social support, teacher social support, and peer social support. The models were initially run including the following covariates, selected based on theoretical reasons: child age, minority status, gender, perceived life threat, and actual life threat. Models were trimmed to exclude nonsignificant paths (*p* ≥ .10) in the final models, as was the case in the Platt et al. (2016) analysis. After trimming nonsignificant paths, if the model exhibited poor fit, modification indices and studentized residuals were consulted to identify relationships that, if modelled, would improve fit. This was done until good model fit was achieved. It resulted in the addition of three statements in the parent model, four statements in the teacher model, and five statements in the peer model. For the primary analyses, the alpha level for statistical significance was defined as *p* < .05.

## 2. Results

### 2.1. Descriptive statistics

Approximately one-quarter of the participants (26%) responded affirmatively to the perceived life-threatening experience item (i.e. ‘Did you think you might die?’). The mean score for actual life-threatening experiences was 0.70 (*SD* = 1), similar to other research of children affected by hurricanes (La Greca et al., 2010; Lai et al., 2013). Table 1 presents mean scores for parent, teacher, and peer social support as well as PTSS scores. Tables 2–4 present bivariate correlations between the main independent and dependent variables.

**Table 2.** Bivariate correlation matrix for parent social support and PTSS variables (*N* = 426).

	Parent SS				PTSS		
	Wave 1	Wave 2	Wave 3	Wave 4	Wave 1	Wave 2	Wave 3
Parent SS							
Wave 2	0.41	1.00					
Wave 3	0.40	0.63	1.00				
Wave 4	0.26	0.50	0.56	1.00			
PTSS							
Wave 1	−0.22	−0.20	−0.27	−0.14	1.00		
Wave 2	−0.12	−0.21	−0.27	−0.20	0.57	1.00	
Wave 3	−0.16	−0.19	−0.36	−0.18	0.52	0.64	1.00
Wave 4	−0.12	−0.12	−0.26	−0.24	0.49	0.56	0.67

**Table 3.** Bivariate correlation matrix for teacher social support and PTSS variables (*N* = 426).

	Teacher SS				PTSS		
	Wave 1	Wave 2	Wave 3	Wave 4	Wave 1	Wave 2	Wave 3
Teacher SS							
Wave 2	0.26	1.00					
Wave 3	0.32	0.53	1.00				
Wave 4	0.27	0.44	0.53	1.00			
PTSS							
Wave 1	−0.14	−0.08	−0.13	−0.16	1.00		
Wave 2	−0.16	−0.14	−0.18	−0.20	0.57	1.00	
Wave 3	−0.18	−0.14	−0.22	−0.21	0.52	0.64	1.00
Wave 4	−0.12	−0.11	−0.21	−0.23	0.49	0.56	0.67

SS = Social Support. PTSS = Posttraumatic Stress Symptoms. Statistically significant correlations are in bold (*p* < .05).

**Table 4.** Bivariate correlation matrix for peer social support and PTSS variables (*N* = 426).

	Peer SS				PTSS		
	Wave 1	Wave 2	Wave 3	Wave 4	Wave 1	Wave 2	Wave 3
Peer SS							
Wave 2	0.50	1.00					
Wave 3	0.47	0.60	1.00				
Wave 4	0.50	0.54	0.65	1.00			
PTSS							
Wave 1	−0.25	−0.20	−0.23	−0.14	1.00		
Wave 2	−0.20	−0.26	−0.35	−0.25	0.57	1.00	
Wave 3	−0.17	−0.29	−0.44	−0.24	0.52	0.64	1.00
Wave 4	−0.29	−0.34	−0.34	−0.36	0.49	0.56	0.67

SS = Social Support. PTSS = Posttraumatic Stress Symptoms. Statistically significant correlations are in bold (*p* < .05).

### 2.2. Social support and PTSS models

**Figure 1.**
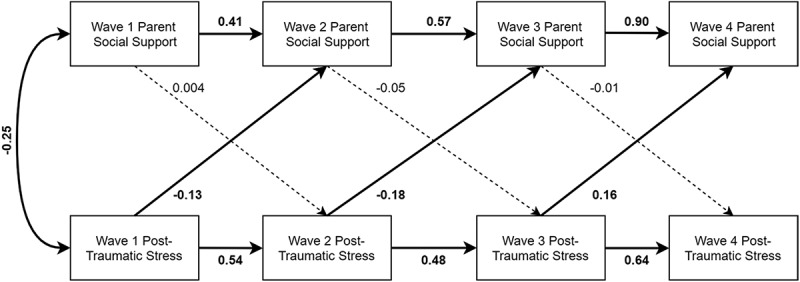
Parent social support model with standardized estimates presented. Relationships with covariates (child age, minority status, gender, perceived life threat, and actual life threat) not included to reduce clutter. Significant paths (*p* < .05) are bolded, and nonsignificant paths are dashed lines.

All final models exhibited good fit according to global fit indices (see Table 5 for final model fit statistics). All paths from social support in one wave to social support in the next immediate wave were statistically significant, as were the paths from one wave of PTSS to the next wave. In the parent social support model, the path from Wave 1 PTSS to Wave 2 social support (standardized estimate = −0.13, 95% CI [−0.24, −0.03], *p* = .02), controlling for child age and Wave 1 social support; from Wave 2 PTSS to Wave 3 social support (standardized estimate = −0.18, 95% CI [−0.26, −0.10], *p* < .0001), controlling for age, minority status, and Wave 2 social support; and from Wave 3 PTSS to Wave 4 social support (standardized estimate = 0.16, 95% CI [0.05, 0.28], *p* = .005), controlling for child gender, actual life threat, and Wave 3 social support were all statistically significant (see Figure 1). No paths from social support to PTSS were statistically significant in the parent model.

**Table 5.** Final model fit statistics (*N* = 426).

Social Support Model	χ2(*df*), *p*-value	RMSEA (90% CI)	CFI	SRMR
Parent	45.25(32), 0.06	0.03 (0, 0.05)	0.99	0.03
Teacher	31.23(24), 0.15	0.03 (0, 0.05)	0.99	0.03
Peer	26.92(17), 0.06	0.04 (0, 0.06)	0.99	0.03

RMSEA = root mean square error of approximation, CI = confidence interval, CFI = comparative fit index, SRMR = standardized root mean square residual. Degrees of freedom are different in each model due to a differing number of covariates included in the models.

**Figure 2.**
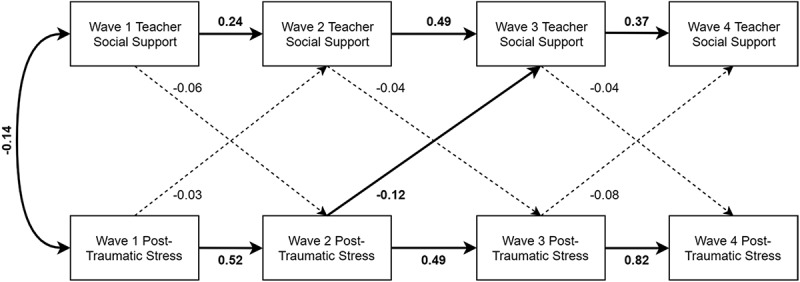
Teacher social support model with standardized estimates presented. Relationships with covariates (child age, minority status, gender, perceived life threat, and actual life threat) are reported in text only to reduce clutter. Significant paths (*p* < .05) are bolded, and nonsignificant paths are dashed lines.

In the teacher social support model, the path from Wave 2 PTSS to Wave 3 social support was statistically significant (standardized estimate = −0.12, 95% CI [−0.22, −0.03], *p* = .009), controlling for gender, actual life threat, and Wave 2 social support. However, other paths between PTSS and social support were nonsignificant (see Figure 2).

**Figure 3.**
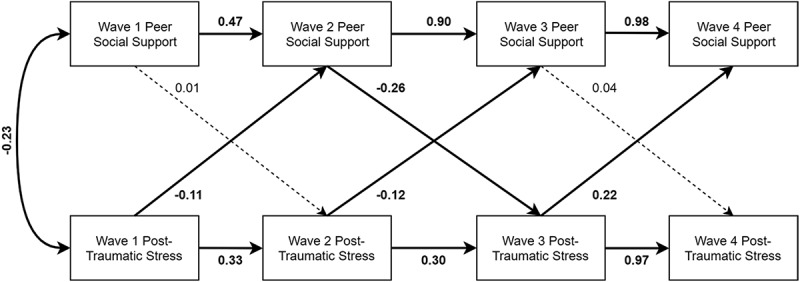
Peer social support model with standardized estimates presented. Relationships with covariates (child age, minority status, gender, perceived life threat, and actual life threat) are reported in text only to reduce clutter. Significant paths (*p* < .05) are bolded, and nonsignificant paths are dashed lines.

Finally, the peer social support model included several statistically significant paths. The path between Wave 1 PTSS to Wave 2 social support (standardized estimate = −0.11, 95% CI [−0.20, −0.02], *p* = .02), controlling for Wave 1 social support; between Wave 2 PTSS to Wave 3 social support (standardized estimate = −0.12, 95% CI [−0.23, −0.01], *p* = .03), controlling for Wave 2 social support; and between Wave 3 PTSS to Wave 4 social support (standardized estimate = 0.22, 95% CI [0.09, 0.35], *p* = .001), controlling for perceived life threat and Wave 3 social support were all significant. Additionally, the path between Wave 2 social support to Wave 3 PTSS (standardized estimate = −0.26, 95% CI [−0.34, −0.17], *p* < .0001), controlling for perceived life threat and Wave 2 PTSS, was statistically significant in the peer model (see Figure 3).

## 3. Discussion

The purpose of this study was to examine the bidirectional relationships between social support (from parents, teachers, and peers) and PTSS among children exposed to Hurricane Katrina. Levels of social support in this sample were similar to that obtained in other studies (La Greca, Lai, Joormann, Auslander, & Short, 2013; La Greca, Silverman, Vernberg, & Prinstein, 1996). To our knowledge, this is the first study to test social causation and social selection models simultaneously among children longitudinally after a disaster. Overall, findings supported the social selection model, and demonstrated limited support for social causation models. Specific findings are discussed below.

Regarding social selection mechanisms (i.e. those with greater PTSS either avoid sources of social support, are selected out of supportive relationships, or perceive less support to be available), we found strong evidence for these mechanisms over the first two years post-disaster (i.e. Waves 1–3 in this study). Listed in order of the source of social support, PTSS predicted decreased parent social support at Waves 2 and 3. PTSS also predicted decreased teacher social support at Wave 3. In addition, PTSS predicted decreased peer social support at Waves 2 and 3. These findings tentatively suggest that PTSS may impact multiple sources of social support for children two years after a disaster. This finding highlights the negative effects that PTSS may have on children in the immediate to long-term recovery period. More specifically, findings provide initial evidence that child PTSS has limited impact on perceptions of teacher support, but PTSS do impact children’s perceptions of parent and peer social support. The findings were strongest for parent and peer support. Although research consistently indicates that children’s social support post-disaster originates from a variety of sources (e.g. parents, peers, teachers, and other community resources, such as mental health professionals), the literature is mixed on how each of these sources differentially impact PTSS in children and vice versa (for a review, see Pfefferbaum, Jacobs, Houston, & Griffine, 2015).

Differences related to sources of social support may be partly due to differences in how social support was assessed. Items measuring parent and peer social support emphasized the child’s perceptions. For example, parent social support included items about whether their parent understood or wanted to hear about their problems. Peer forms of social support included items about whether they had a friend who understood them, who they could talk to, and with whom they spend time. In contrast, children rated teacher support in a more general manner. For example, items included whether the child perceived their teachers as fair or that they treated them like a person. Thus, findings indicate that PTSS may have limited impact on global assessments of others’ support, but does impact specific, individual perceptions of support. Future studies are also needed that will test whether social selection mechanisms might exist due to PTSS causing children to select out of relationships or to change their perceptions of support.

Surprisingly, Wave 3 PTSS predicted increased social support from parents and peers at Wave 4. This was unexpected. It suggests that perhaps prolonged distress (i.e. PTSS at Wave 3) may trigger parents and peer recognition of the significance of distress. Furthermore, it is possible that higher social support at Wave 4 could be related to parents’ and peers’ recovery post-disaster. The parents also experienced post-disaster-related distress, which may have impacted children’s recovery (Cobham, McDermott, Haslam, & Sanders, 2016; Kelley et al., 2010; Self-Brown, Lai, Harbin, & Kelley, 2014; Spell et al., 2008). It is possible that during the first two-years post-disaster, parents and peers are recovering themselves, thus decreasing the availability of social support for others.

Additionally, in the immediate aftermath of a natural disaster, families and communities are focused on meeting basic needs such as access to clean water and rebuilding schools and homes (Weems & Overstreet, 2008). According to estimates from a representative sample of pre-Katrina residents, approximately 42% of individuals were still displaced 14 months following the disaster (Fussell, Sastry, & VanLandingham, 2010). Following Hurricane Katrina, there were significant concerns regarding access to clean water and other environmental hazards that required intervention of various local, state, and federal agencies (Manuel, 2006). Further, approximately 55% of public schools remaining closed two years following the event (Liu & Plyer, 2007). Due to the significant disruptions in availability of these basic resources, parents may have been focused on restoring consistency and stability for their families, reducing the availability of social support they may have been able to provide to others. As time elapses and basic needs are met, parents may be better equipped to provide adequate social support to their children. This might explain why child PTSS was associated with higher levels of support two years post-disaster. This explanation underscores an ecological needs-based perspective when assessing mental health outcomes in children exposed to traumatic events (Bronfenbrenner, 1994; Sandler, 2001; Weems & Overstreet, 2008). Further, it is possible that children may have experienced additional trauma before Wave 3 which triggered parent and peer support; however, this was not assessed in this study.

With regard to social causation models (i.e. those with less social support are at increased risk for PTSS), our study found only limited support for these mechanisms. Specifically, no relationships between parent or teacher social support to subsequent PTSS were found. However, peer social support at Wave 2 was negatively related to Wave 3 PTSS. In the context of the larger literature on disasters, these findings are somewhat surprising. Multiple studies have found that social support is related to PTSS among children exposed to disasters (e.g. Moore & Varela, 2010). Banks and Weems (2014) found parent and peer social support to predict distress longitudinally and concurrently among youth exposed to disaster. However, the majority of studies examining how social support impacts youth distress post-disaster are correlational and retrospective, and do not include bidirectional models. As an example, Bokszczanin (2008) found that parent social support predicted levels of PTSS among adolescents 28 months after a flood. Assessments in that study were completed at one timepoint only.

Our findings for limited support for social causation models are similar to those found in other longitudinal studies. Platt et al. (2016) found very limited evidence for social causation mechanisms among adults. Specifically, they found that emotional support at 2–6 months post-disaster was related to PTSS at 5–9 months post-disaster, but they found no significant relationships at their later timepoint (i.e. 14–19 months post-disaster). Further, Blanc, Bui, Mouchenik, Derivois, and Birmes (2015) assessed children exposed to the 2010 Haiti earthquake. They examined the effect of psycho-social support on an intervention group versus a control. They found no difference in average PTSD scores between the psycho-social support group and controls. However, they noted that the two groups were not equivalent in terms of socio-demographics. Taken together with our findings, this suggests that social support may have limited impact on PTSS beyond the first two years post-disaster. Though speculation, it is possible that the multiple resource and housing needs of families during the long-term recovery period supersede needs for social support.

There are several limitations to the current paper. First, all data were based on child self-report. Evidence suggests there is low concordance between parent and child reports post-disaster (Lai, Beaulieu, Ogokeh, Self-Brown, & Kelley, 2015a). Further, the timing of assessments was variable. Although we followed standard practice and selected the midpoint of each assessment (e.g. Brown et al., 2016), this variation limits the generalizability of our study and its implications for disaster interventions. In addition, our initial assessment was 3–7 months after Hurricane Katrina, a time period beyond the immediate aftermath of the disaster, when supportive interventions are usually deployed. Future studies are needed that include more immediate assessments in order to better understand the relationship between social support and PTSS over time. Next, our sample of children was exposed to Hurricane Katrina. In many ways, Katrina was a unique extreme weather event, in that many children were already vulnerable in New Orleans and the surrounding areas before the disaster. This is a strength from the perspective of learning about complex trauma in vulnerable populations, but findings may not translate to other disasters. Additionally, as is often the case in longitudinal studies, there was some attrition in the sample with 61 (14%) participants dropping out of the study by Wave 3. However, when we compared those who completed the study versus those who dropped out, we found no significant differences. Further, while the percent of participants who reported a perceived life threatening experience during the hurricane was similar to previous research (Hensley & Varela, 2008), the mean reported actual life threatening experiences was low in this sample. The low exposure in this sample may indicate that their exposure was peripheral. Findings for those in our sample may differ from experiences of children with more direct exposure to a disaster. In addition, our study only included children who were in school. This may limit the generalizability of our findings, given that the most vulnerable children were likely those who did not return to schools, who were displaced from their homes (i.e. residing in temporary trailers or shelters), or who had to move away from the New Orleans area (Hansel, Osofsky, Osofsky, & Friedrich, 2013; Osofsky & Osofsky, 2013; Osofsky, Osofsky, Kronenberg, Brennan, & Hansel, 2009).

Despite these limitations, the findings have important implications for research and practice. Our research builds on previous literature highlighting the impact and complexity of the relationship between social support and PTSS in children’s recovery from a natural disaster (e.g. La Greca, Silverman, Lai, & Jaccard, 2010). The findings suggest that PTSS generally precede decreases in social support for children. If other studies with more frequent assessments and less variability in assessment timing replicate these results, this would add to emerging literature suggesting that mental health symptoms should be the primary target for children after disasters. This is not to suggest that promoting social support and connection are not critical post-disaster. Rather, our findings provide initial support that in cases of limited funding, PTSS should be prioritized, given their cascading effects on social support.
